# pH-Responsive Host-Guest Complexations Between a Water-Soluble Pillar[6]arene Dodecyl-Ammonium Chloride and Aromatic Sulfonic Acids

**DOI:** 10.3389/fchem.2020.588201

**Published:** 2020-09-15

**Authors:** Qunpeng Duan, Fei Wang, Hongsong Zhang, Kui Lu

**Affiliations:** ^1^Henan International Joint Laboratory of Rare Earth Composite Materials, School of Materials and Chemical Engineering, Henan University of Engineering, Zhengzhou, China; ^2^School of Chemical Engineering and Food Science, Zhengzhou Institute of Technology, Zhengzhou, China

**Keywords:** pillararenes, supramolecular chemistry, binding motif, pH-responsiveness, aromatic sulfonic acids

## Abstract

In the present work, new host-guest binding motifs based on a water-soluble pillar[6]arene dodecyl-ammonium chloride (CP6) with two aromatic sulfonic acids in aqueous media were fabricated. In accordance with the integrated results of ^1^H NMR, 2D NOESY, and florescence titration experiments, it was demonstrated that the host-guest binding of CP6 with the two aromatic sulfonic acids in aqueous solution not only has high binding constants but also has pH-responsiveness.

## Introduction

In the supramolecular chemistry field, stimuli-responsive molecular host-guest recognition motifs are attracting much attention because of their wide range of applications in the fabrication of various fascinating and important supramolecular systems, such as molecular devices and machines (Palmer and Rebek, 2004; Han and Chen, 2007; Zhang et al., 2014), responsive supramolecular polymers (Xu et al., 2013; Cantekin et al., 2015), and other smart supramolecular materials (Avestro et al., 2012; Guo and Liu, 2012; Li et al., 2012; Vukotic and Loeb, 2012). Up to now, pH, temperature, light, redox reagents, enzymes, and other external stimuli have been widely utilized for the fabrication of various responsive host-guest complexation systems (Guo and Liu, 2014; Han et al., 2014; Ma and Tian, 2014). Among these stimuli, pH response is very interesting for special applications in electronic devices, gene delivery, and drug delivery (Credit et al., 1997; Badjic et al., 2004; Yu et al., 2012; Duan et al., 2013; Zhang et al., 2013). Therefore, it is of particular importance to construct pH-responsive molecular host-guest complexation systems.

**Scheme 1:**
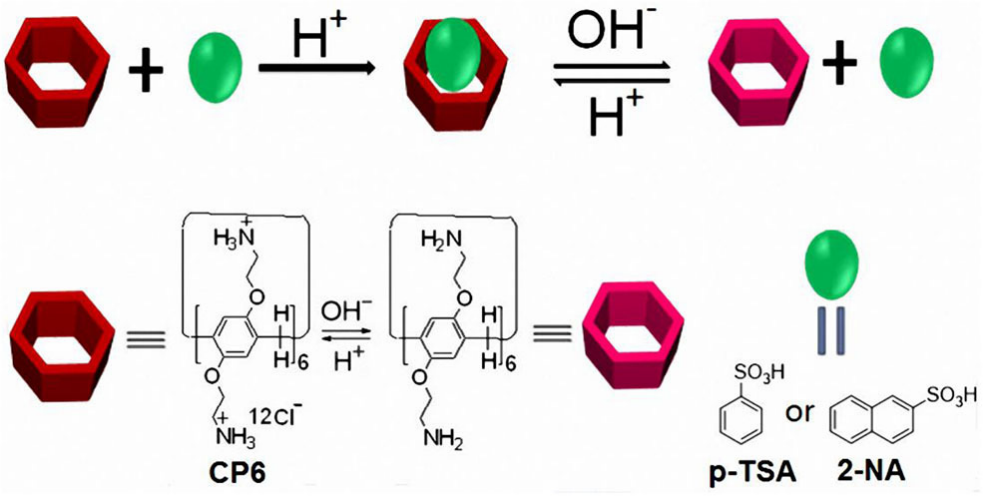
Chemical structures and cartoon representation of pillararene **CP6** and guests (**p-TSA** and **2-NA)**; illustration of the acid/base controlled dethreading/rethreading process.

Pillararenes (Ogoshi et al., 2008, 2016; Cao et al., 2009, 2014; Xue et al., 2012; Yao et al., 2012; Si et al., 2014; Wang et al., 2019), as a new kind of supramolecular macrocyclic hosts, have gained growing attention due to their intrinsic unique rigid and symmetrical pillar-shaped architecture, tunable cavity size, easy modification, and superior host-guest properties. Pillararenes endowed with these outstanding features have been used to construct numerous supramolecular systems, such as supramolecular polymers (Zhang et al., 2011; Guan et al., 2012; Li, 2014), daisy chains (Zhang et al., 2012), transmembrane channels (Si et al., 2014), drug-release systems (Cao et al., 2014; Chang et al., 2014; Hu et al., 2016), and other advanced functional materials (Ni et al., 2016; Wang et al., 2018; Xiao et al., 2018; Zhou et al., 2020). Practically, a series of water-soluble pillararenes have been synthesized and demonstrated to act as scaffolding hosts to various guests (Ogoshi et al., 2012; Hu et al., 2016; Yakimova et al., 2016). Among these water-soluble pillararenes, pH-responsive ones have been reported in the construction of plenty of supramolecular systems (Yu et al., 2012; Cao et al., 2014; Hu et al., 2016; Xiao et al., 2019). Recently, a pillar[6]arene dodecyl-ammonium chloride (**CP6**) with good water solubility was prepared by our group (Duan et al., 2019). The **CP6** with 12 –${\text{NH}}_{3}^{+}$ groups on both rims has response to acid/base reagent pairs (such as HCl and NaOH). Searching new guests for this positively charged pillar[6]arene to fabricate pH-responsive host-guest binding motifs is thus of great interest. In the present manuscript, two aromatic sulfonic acids, i.e., p-toluenesulfonic acid (**p-TSA**) and 2-naphthalenesulfonic acid (**2-NA**) were selected as guests, owing to their wide uses in rubbers, dyestuffs, insecticides, varnishes, and pharmaceuticals (Wu et al., 2011). The design and fabrication of pH-responsive host-guest recognition motifs are elaborated in Scheme 1. We show that **CP6** can form highly stable host-guest complexes (**p-TSA**⊂**CP6** and **2-NA**⊂**CP6**) with **p-TSA** and **2-NA**, respectively. Based on these two molecular recognition motifs, pH-responsive host-guest complexes were demonstrated.

## Materials and Methods

All reagents were commercially available and used as supplied without further purification. **CP6** (Duan et al., 2019) was prepared according to the published procedures. NMR spectra were conducted on Bruker Avance III HD 400 spectrometer with the use of the deuterated solvent as the lock and the residual solvent as the internal reference. Fluorescence spectra were performed on an Agilent Cary Eclipse fluorescence spectrophotometer. 0.1 M phosphate buffer solution (PBS, pH = 6.0) was prepared by mixing 12 mL 1 M Na_2_HPO_4_ and 88 mL 1 M NaH_2_PO_4_ solution. The D_2_O solutions were adjusted to pD 6.0 by DCl or NaOD. The pH and pD values were verified on a Mettler Toledo pH meter calibrated with two standard buffer solutions. pH readings were converted to pD by adding 0.4 units (Glasoe and Long, 1960).

## Results and Discussion

**Figure 1:**
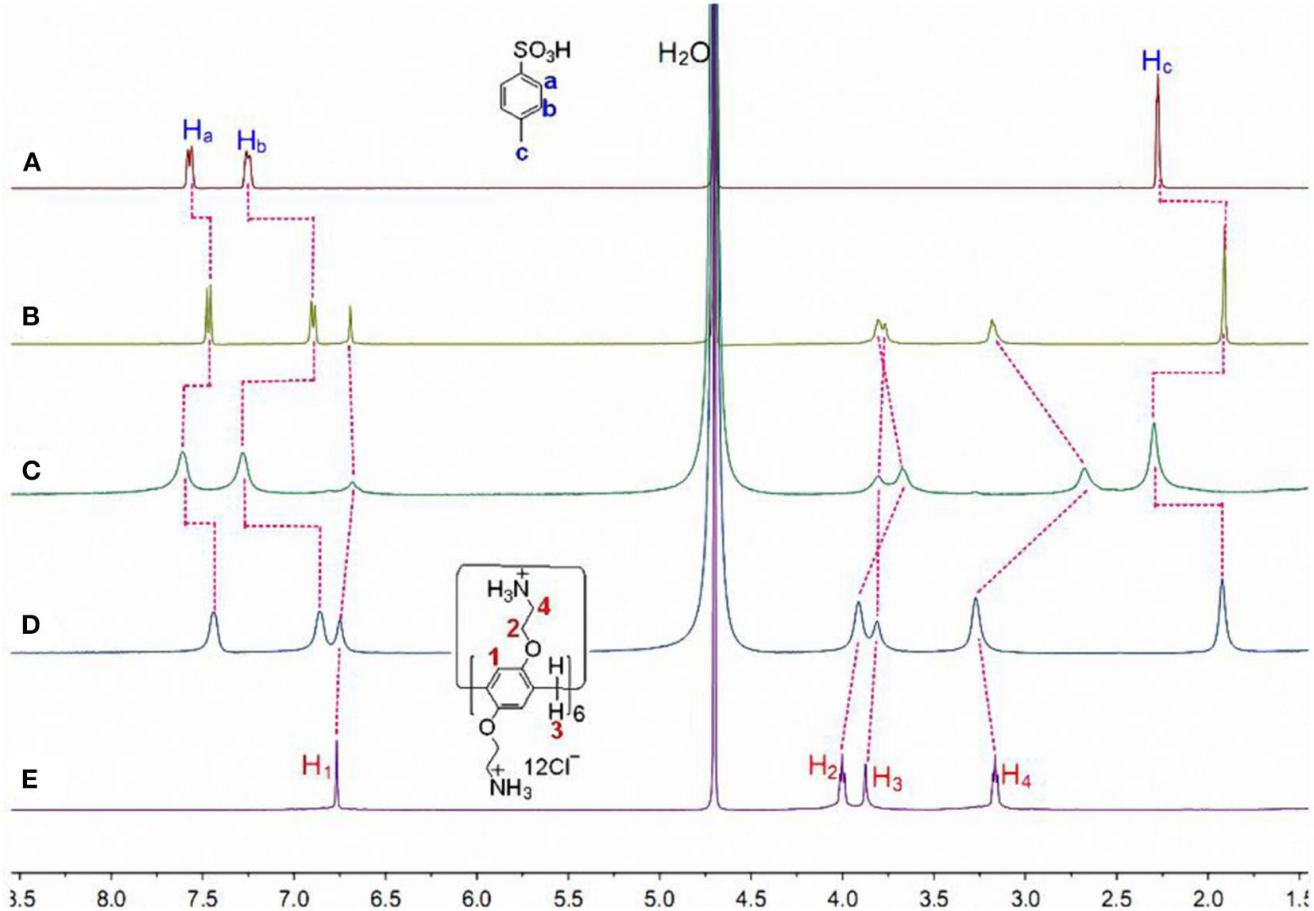
Partial ^1^H NMR spectra (400 MHz, D_2_O, 298 K) of **(A)** 20.00 mM **p-TSA**, **(B)** 2.00 mM **CP6** and 20.00 mM **p-TSA**, **(C)** after addition of 1.0 μL of aqueous NaOD solution (30%) to b, **(D)** after addition of 2.0 μL of aqueous DCl solution (20%) to c, and **(E)** 2.00 mM **CP6** at pD 6.0.

**Figure 2:**
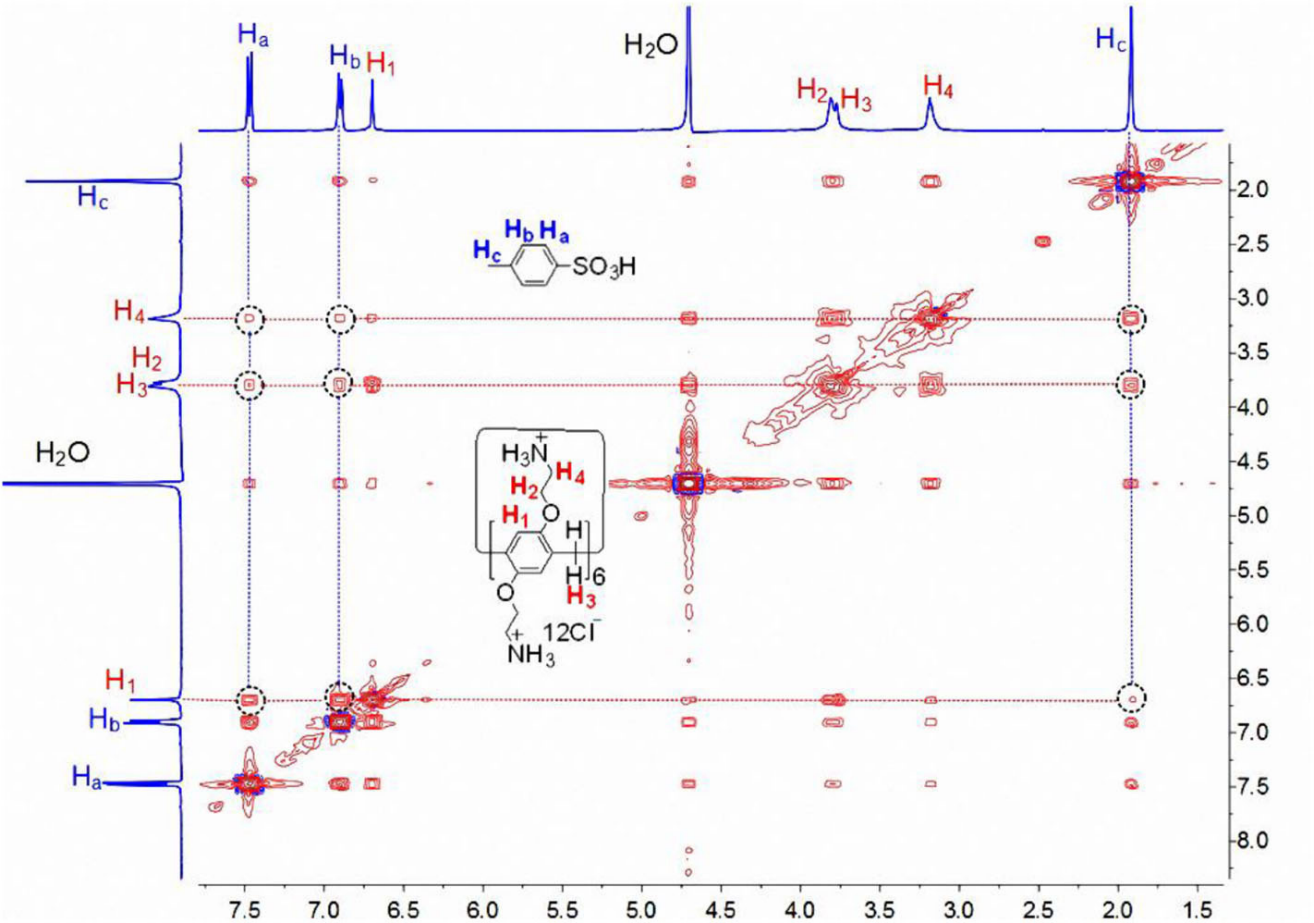
2D NOESY NMR (400 MHz, D_2_O, 298 K, mixing time = 300 ms) spectrum of a solution of **CP6** (2.00 mM) and **p-TSA** (20.00 mM).

The host-guest complexation of **CP6** with **p-TSA** in D_2_O was first investigated by ^1^H NMR spectroscopy. As shown in Figure 1B, when adding about 0.1 equiv. of the host **CP6**, the signals of protons (a-c) on the guest **p-TSA** demonstrate significant upfield shifts against free guest **p-TSA** proton signals (Δδ = −0.15, −0.39, and −0.37 ppm for proton a, b and c, respectively). Strong upfield chemical shifts (Δδ) of the aromatic and methyl protons indicate that the **p-TSA** guest was fully threaded into the host cavity, forming a stable threaded host-guest complex. And the presence of only one set of peaks for the solution of **CP6** and **p-TSA** (Figure 1B) suggests that the host-guest complex formation is a fast exchange process on the NMR time scale. The host-guest binding of **CP6** and **p-TSA** in water was then examined by 2D NOESY analysis. From the 2D NOESY spectrum (Figure 2), NOE correlation signals were observed between H_a_, H_b_, and H_c_ on **p-TSA** and protons H_1−4_ on **CP6**, respectively, supporting the assignment of a threaded structure **p-TSA**⊂**CP6**.

**Figure 3:**
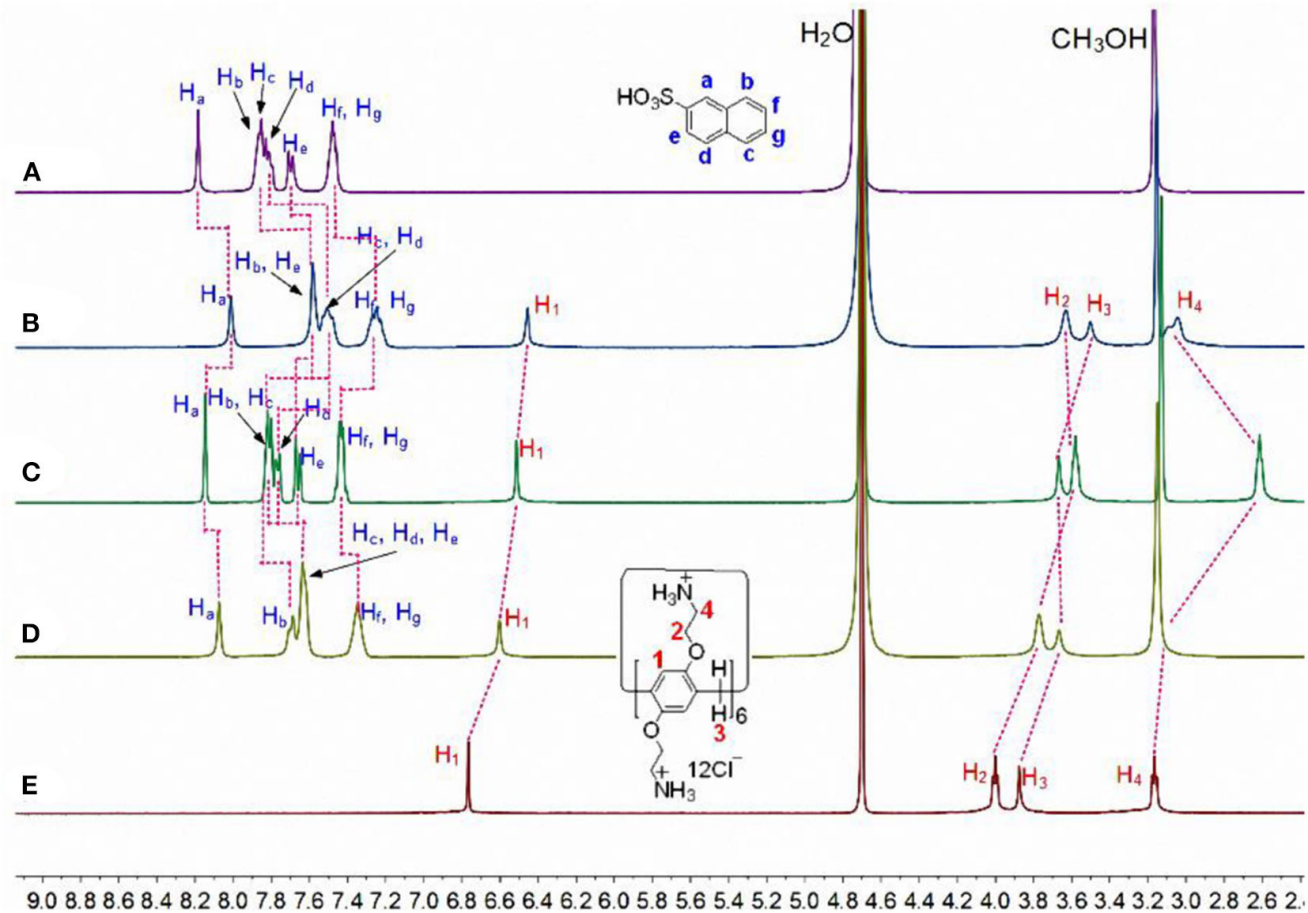
Partial ^1^H NMR spectra (400 MHz, D_2_O/CD_3_OD = 1/1, v/v, 298 K) of **(A)** 20.00 mM **2-NA**, **(B)** 2.00 mM **CP6** and 20.00 mM **2-NA**, **(C)** after addition of 1.0 μL of aqueous NaOD solution (30%) to b, **(D)** after addition of 2.0 μL of aqueous DCl solution (20%) to c, and **(E)** 2.00 mM **CP6** at pD 6.0.

**Figure 4:**
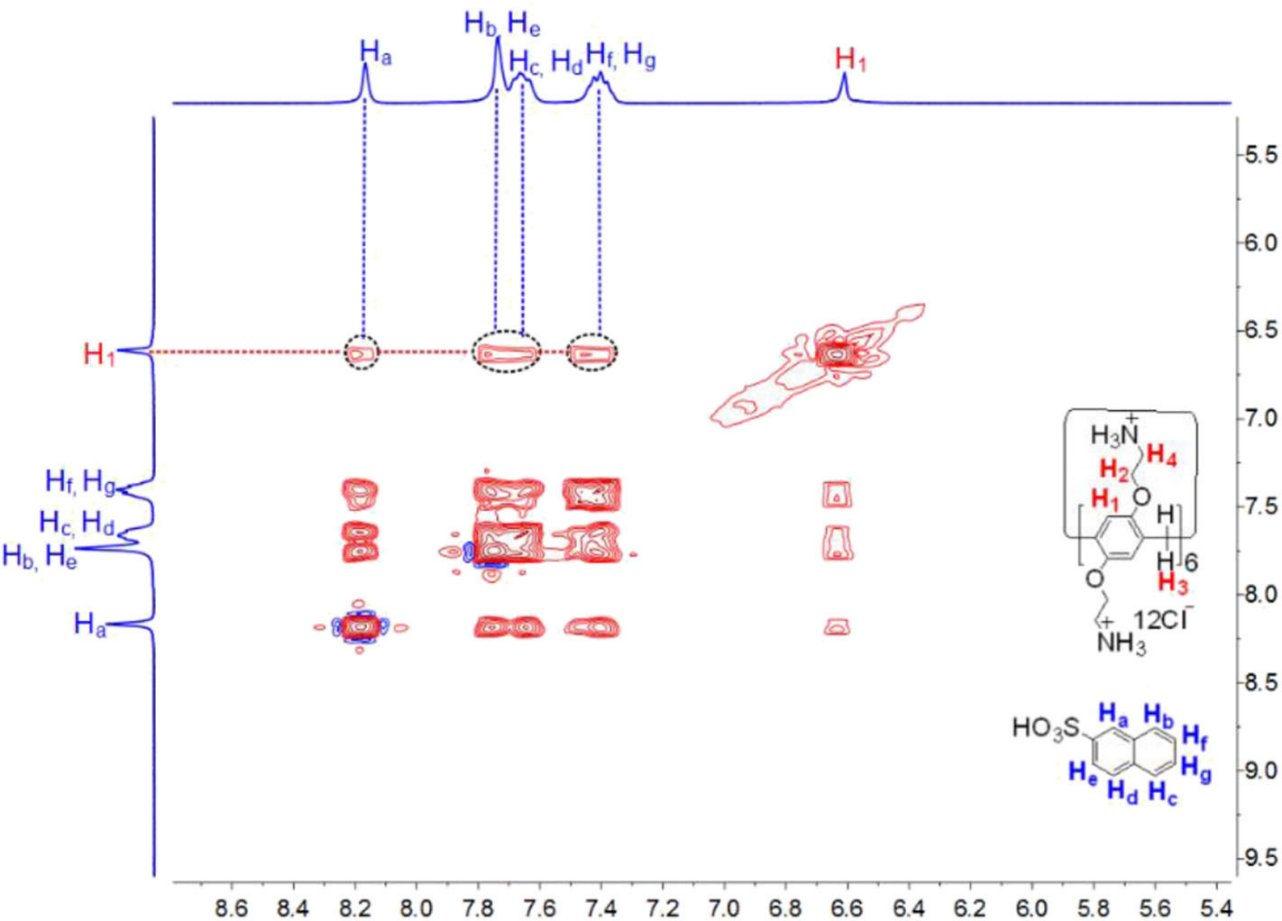
2D NOESY NMR (400 MHz, D_2_O, 298 K, mixing time = 300 ms) spectrum of a solution of **CP6** (2.00 mM) and **2-NA** (20.00 mM).

Subsequently, the complexation of **CP6** by the larger **2-NA** guest was also investigated. Similarly, ^1^H NMR experiments were also performed to investigate the host-guest complexation of **CP6** with **2-NA** in D_2_O and MeOD. Figures 3A,B show the ^1^H NMR spectra of **2-NA** in D_2_O recorded in the absence and the presence of about 0.1 equiv. of the host **CP6**, respectively. In the presence of **CP6** (Figure 3B), the signals of protons (a-g) on the guest **2-NA** exhibit substantial upfield shifts compared to those of the free **2-NA** (Δδ = −0.24 to −0.16 ppm) (Figure 3A), suggesting the inclusion of the naphthalene moiety of **2-NA** into the hydrophobic **CP6** cavity. The assignment of these naphthyl proton signals of the inclusion complex can be verified by the analysis of the ^1^H-^1^H COZY data (correlation spectroscopy; see Supplementary Figure 1). Furthermore, these shifts appeared due to fast proton exchange observed for complexation in the ^1^H NMR timescale. The 2D NOESY data (Figure 4) show the NOE correlations between the aromatic protons (H_a−g_) of the entrapped **2-NA** and the aromatic proton H_1_ of **CP6**, which also revealed the interpenetrated geometry.

To quantitatively estimate the binding behaviors of **p-TSA** and **2-NA** with host **CP6**, fluorescence titrations were conducted at 298 K in a PBS of pH 6.0. Job plots (Supplementary Figures 2,3) based on the fluorescence titrations data indicated that **CP6** and the two guests form a 1:1 host-guest complex in aqueous solution, respectively. By using a non-linear curve-fitting method (Supplementary Figures 4,5), the association constants (*K*_a_) were calculated to be (2.23 ± 0.15) × 10^4^ M^−1^ and (1.97 ± 0.28) × 10^4^ M^−1^ for **p-TSA** and **2-NA**, respectively. According to the p*K*_a_ values of the two aromatic sulfonic acids (**p-TSA**: −2.1; **2-NA**: −1.8), it can be concluded that the sulfonic groups of the two aromatic sulfonic acids should be in the deprotonated form at pH 6.0. Thus, we conclude that the interaction mechanism of **CP6** with the two aromatic sulfonic acids is that the acidic aromatic sulfonic acids with one sulfonate anion could bind positively charged **CP6** bearing 12 –${\text{NH}}_{3}^{+}$ groups in aqueous solutions at pH 6.0, where the electrostatic interactions between sulfonate anion of the two acidic aromatic sulfonic acids and the cationic portals of the host **CP6** play a dominant role in formation of the present host-guest complexation. By comparing **p-TSA** and **2-NA**, we can investigate the capability of these guests to form host-guest complexes because of the changed size of the hydrophobic part. The *K*_a_ value for **p-TSA** is almost same as that for **2-NA**. Although **2-NA** has one more benzene ring than **p-TSA** and thus the larger π-conjugated system could afford a stronger π-π stacking interaction with the host cavity (Gómez et al., 2014), the electrostatic attractive forces are dominant in these two host-guest complexes.

Additionally, both the obtained p-TSA⊂CP6 and 2-NA⊂CP6 have pH-responsiveness, i.e., the dynamic behavior for the inclusion process of the two aromatic sulfonic acids and CP6 can be reversed upon the addition of HCl and NaOH aqueous solutions. Proton NMR studies were conducted to affirm these two reverse processes (Figures 1, 3). As shown in Figures 1C, 3C, when adding NaOD to the mixed solution of p-TSA⊂CP6 and 2-NA⊂CP6, respectively, both signals of p-TSA and 2-NA returned to almost their uncomplexed positions, suggesting that both p-TSA and 2-NA dethreaded from the cavity of CP6. The reason is apparent: the addition of an aqueous NaOD solution yielded a basic solution and the ${\text{NH}}_{3}^{+}$ groups on CP6 were deprotonated to produce the neutral amino groups, resulting in the disappearance of the electrostatic attractive forces between p-TSA or 2-NA and CP6. However, after adding DCl to these solutions, ^1^H NMR spectra similar to those of the original solutions of p-TSA⊂CP6 and 2-NA⊂CP6 were obtained (Figures 1D, 3D), resulting from the protonation of the amino groups and the regeneration of the complexes between p-TSA or 2-NA and CP6 in this solution. Thus, the host-guest complexes p-TSA⊂CP6 and 2-NA⊂CP6 can be reversed by the sequential addition of a base and an acid (NaOH and HCl, respectively) between its complexed and decomplexed states. In a word, the host-guest complexation between p-TSA or 2-NA and CP6 is pH-responsive and its reversible property can be used to serve as excellent motifs for a variety of controlled molecular release applications.

## Conclusions

In summary, novel pH-responsive host-guest recognition motifs based on a water-soluble pillar[6]arene dodecyl-ammonium chloride **CP6** with **p-TSA** or **2-NA** were successfully constructed. It was established that **CP6** could form a stable 1:1 inclusion complex with the two aromatic sulfonic acids, **p-TSA** and **2-NA**, respectively. Furthermore, both these host-guest complexes can be controlled reversibly through the sequential addition of a base and an acid (NaOH and HCl, respectively) between its complexed and decomplexed states. Consequently, the findings of this work enrich the fields of controlled pillararene chemistry. Our further work will focus on expanding these new pH-responsive host-guest binding motifs to construct smart supramolecular materials in the treatment of sulfonated aromatic pollutants in aqueous media.

## Data Availability Statement

All datasets generated for this study are included in the article/Supplementary Material.

## Author Contributions

QD, FW, and KL designed the work. QD made contributions to the experiments and collective data. The paper was written by QD. All authors extensively discussed the results, reviewed the manuscript, and approved the final version of the manuscript to be submitted.

## Conflict of Interest

The authors declare that the research was conducted in the absence of any commercial or financial relationships that could be construed as a potential conflict of interest.
