# Supplementary material for: pH-Responsive Host-Guest Complexations Between a Water-Soluble Pillar[6]arene Dodecyl-Ammonium Chloride and Aromatic Sulfonic Acids
Source: Front Chem. 2020 Sep 15;8:588201. doi: 10.3389/fchem.2020.588201 (PMC7533581; doi:10.3389/fchem.2020.588201)
Supplement: Supplementary file 1 [file Table_1.DOCX]

Supplementary Material

*1. 2D COSY spectrum of* **2-NA**⊂**CP6** *in D_2_O*


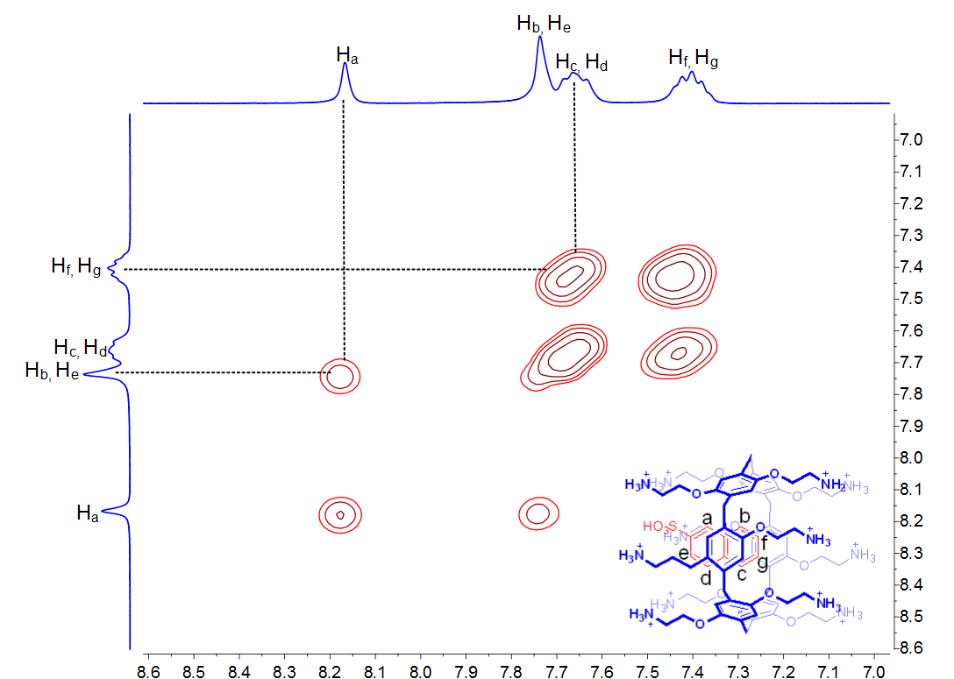


**Supplementary Figure 1.** 2D ^1^H-^1^H COSY spectrum of **2-NA**⊂**CP6** (400 MHz, D_2_O, 298 K), [**CP6**] = 2.00 mM. [**2-NA**] = 20.00 mM.

*2. Determination of the association constants between substrates (****p-TSA, 2-NA****) and* ***CP6***

*2.1 Job plot for* ***p-TSA/2-NA****⊂****CP6***







**Supplementary Figure 2.** (a) Fluorescence spectra of the mixture of **CP6** and **p-TSA** in water at different molar ratios while [**CP6**] + [**p-TSA**] = 1.0 × 10^−5^ M. (b) Job plot showing the 1:1 stoichiometry of the complex between **CP6** and **p-TSA** by plotting the difference in fluorescent emission intensity at *λ*_emission_ = 326 nm (*λ*_excitation_ = 290 nm) against the mole fraction of **p-TSA** at an invariant total concentration of 0.01 mM in aqueous solution.







**Supplementary Figure 3.** (a) Fluorescence spectra of the mixture of **CP6** and 2-**NA** in water at different molar ratios while [**CP6**] + [2-**NA**] = 1.0 × 10^−5^ M. (b) Job plot showing the 1:1 stoichiometry of the complex between **CP6** and 2-**NA** by plotting the difference in fluorescent emission intensity at *λ*_emission_ = 326 nm (*λ*_excitation_ = 290 nm) against the mole fraction of 2-**NA** at an invariant total concentration of 0.01 mM in aqueous solution.

*2.2 Association constants of* ***p-TSA/2-NA****⊂****CP6***

To determine the association constants for the complexation between **CP6** and substrates (**p-TSA** and **2-NA**), fluorescence titration experiments were carried out in a phosphate buffer solution of pH 6.0 which had a constant concentration of **CP6** (1.0 × 10^–5^ M) and varying concentrations of substrates. By a non-linear curve-fitting method, the association constants (*K*_a_) of ***p-TSA/2-NA****⊂****CP6*** were estimated.

The non-linear curve-fittings were based on the equation:

*ΔF* = (*ΔF*_∞_/[H]_0_) (0.5[G]_0_ + 0.5([H]_0_+1/*K*_a_)−(0.5 ([G]_0_^2^+(2[G]_0_(1/*K*_a_ − [H]_0_)) + (1/*K*_a_ + [H]_0_)^2^) ^0.5^)) (eq. 1)

Where *ΔF* is the fluorescence intensity changes at 326 nm at [H]_0_, *ΔF_∞_* is the fluorescence intensity changes at 326 nm when **CP6** is completely complexed, [G]_0_ is the initial concentration of substrates (**p-TSA** and **2-NA**), and [H]_0_ is the fixed initial concentration of **CP6**.^S1^







**Supplementary Figure 4.** (a) Fluorescence spectra of **CP6** (1.0 × 10^–5^ M) upon addition of **p-TSA** (0-3.71 × 10^–4^ M) in aqueous solution (excited at 290 nm) at room temperature. Upon addition of **p-TSA**, emission from **CP6** was quenched, indicating the formation of the **p-TSA**⊂**CP6** complex. (b) The fluorescence intensity changes of **CP6** upon addition of **p-TSA**. The red solid line was obtained from the non-linear curve-fitting using eq. 1.







**Supplementary Figure 5.** (a) Fluorescence spectra of **CP6** (1.0 × 10^–5^ M) upon addition of 2-**NA** (0-3.71 × 10^–4^ M) in aqueous solution (excited at 290 nm) at room temperature. Upon addition of 2-**NA**, emission from **CP6** was strengthened, indicating the formation of the 2-**NA**⊂**CP6** complex. (b) The fluorescence intensity changes of **CP6** upon addition of **2-NA**. The red solid line was obtained from the non-linear curve-fitting using eq. 1.

*References:*

- S1. (a) K. A. Connors, Binding Constants, Wiley: New York, 1987. (b) P. S. Corbin, Ph.D. *Dissertation*, University of Illinois at Urbana-Champaign, Urbana, IL, 1999. (c) P. R. Ashton, R. Ballardini, V. Balzani, M. Belohradsky, M. T. Gandolfi, D. Philp, L. Prodi, F. M. Raymo, M. V. Reddington, N. Spencer, J. F. Stoddart, M. Venturi, D. J. Williams, *J. Am. Chem. Soc.* 118 (1996) 4931−4951. (d) J. Zhang, F. Huang, N. Li, H. Wang, H. W. Gibson, P. Gantzel, A. L. Rheingold, *J. Org. Chem.* 72 (2007) 8935−8938.
